# Preconditioning the uterine unfolded protein response maintains non-apoptotic Caspase 3-dependent quiescence during pregnancy

**DOI:** 10.1038/s41419-018-1000-4

**Published:** 2018-09-17

**Authors:** Judith Ingles, Arren Simpson, Chandrashekara Kyathanahalli, Prashanth Anamthathmakula, Sonia Hassan, Pancharatnam Jeyasuria, Jennifer C. Condon

**Affiliations:** 1 Department of Physiology, School of Medicine, Wayne State University, Detroit, MI USA; 2 Department of Biology, University of Detroit Mercy, Detroit, MI USA; 3 Department of Obstetrics and Gynecology, Wayne State University, Detroit, MI USA; 4 Perinatal Research Initiative in support of the Perinatology Research Branch, Division of Obstetrics and Maternal-Fetal Medicine, Division of Intramural Research, Eunice Kennedy Shriver National Institute of Child Health and Human Development, National Institutes of Health, U. S. Department of Health and Human Services, Bethesda and Detroit, MD and MI USA

## Abstract

The prevention of apoptotic caspase 3 activation through biological preconditioning, mediated through the modulation of the unfolded protein response has been demonstrated to ameliorate multiple pathophysiologies. The maintenance of non-apoptotic caspase 3 activity by the unfolded protein response within the pregnant uterus has previously been proven to be critical in inhibiting uterine myocyte contractility during pregnancy. Here we report that the pregnant uterus utilizes an unfolded protein response-preconditioning paradigm to conserve myometrial caspase 3 in a non-apoptotic state in order to effectively inhibit uterine contractility thereby preventing the onset of preterm labor. In the absence of appropriate endogenous preconditioning during pregnancy, uterine caspase 3 is transformed from a non-apoptotic to an apoptotic phenotype. Apoptotic caspase 3 activation results in the precocious triggering of local uterine inflammatory signaling and prostaglandin production, consequently resulting in an increased incidence of preterm birth. These findings represent a paradigm shift in our understanding of how preconditioning promotes the maintenance of uterine non-apoptotic caspase 3 action during pregnancy preventing the onset of premature uterine contraction and therefore defining the timing of the onset of labor.

## Introduction

Activation of caspase 3 (CASP3) is typically a hallmark of cellular apoptosis or programmed cell death. Conversely, within the pregnant uterine myocyte our laboratory has established that CASP3 activation via the unfolded protein response (UPR) is completely non-apoptotic in nature and plays a critical role in the regulation of uterine myocyte quiescence^1,2^. While the tocolytic action of myometrial CASP3 has been highly characterized, it remains elusive as to how the uterus capacitates active CASP3 in a non-apoptotic state during pregnancy. A growing body of evidence suggests that non-apoptotic CASP3 action can be maintained through the process of cellular preconditioning^3–5^. Throughout pregnancy, it is well established that the uterus experiences various biochemical and physiological stimuli that have been shown to trigger the UPR in other biological systems. For example, following fertilization the myometrium experiences hyperplasia^6^, hypertrophy^7^, hypoxia^8^, hormone fluctuations^9^, inflammation^10^, and mechanical stretch^11^ all of which have been demonstrated to activate the UPR. Subsequently, we hypothesize that incremental endoplasmic reticulum stress insults experienced by the uterus during gestation act to capacitate the myometrium to withstand additional more severe stressors, while maintaining the tocolytic action of non-apoptotic CASP3, thus preventing premature uterine contractility.

Classically, CASP3 activity has been linked to the execution of cellular apoptosis through proteolytic cleavage of DNA repair molecules, such as poly ADP-ribose polymerase (PARP), resulting in  internucleosomal cleavage and fragmentation of DNA. In the myometrium however, UPR-dependent activation of dna damage inducible transcript 3 (GADD153) has been demonstrated to maintain active non-apoptotic CASP3 at high levels throughout early and mid-gestation^12^. In other muscle systems such as the bladder, heart, and diaphragm non-apoptotic CASP3 has been described to have anti-contractile function^13–15^. Similarly, we have shown in the uterus CASP3 inhibits myometrial contraction through the targeted cleavage and degradation of multiple components of the contractile architecture, i.e., connexin 43, α-actin, and γ-actin^1,2^. While the tocolytic function of CASP3 is imminently important, understanding how the pregnant uterus maintains active CASP3 is equally important and may elucidate novel therapeutic targets for inhibiting preterm labor.

Biological preconditioning refers to the phenomenon in which prophylactic insults afford cellular/tissue tolerance against ensuing damaging or lethal stress insults, largely mediated through modulation of the local cellular UPR, allowing for enhanced cell survival resulting in a heightened resistance to apoptotic cell death^16^. In the events of neuronal ischemic preconditioning specifically, transient hypoxic episodes have been demonstrated to reduced cellular apoptosis while maintaining active non-apoptotic CASP3 expression following sustained ischemic injury^5^. As previously mentioned, the myometrium experiences various endogenous modalities of stress, i.e., hyperplasia, hypertrophy, hypoxia, hormonal fluctuations, and mechanical stretch without undergoing apoptosis, that have been demonstrated in other organ systems to illicit an UPR. In a pregnant mouse model, our laboratory has demonstrated that alteration of uterine adaptive UPR signals, i.e., glucose regulated protein (GRP78) and tocolytic CASP3 action significantly modifies gestational length^1^. Specifically, precocious exaggerated uterine stress prematurely increases adaptive GRP78 signaling, eliminating the tocolytic action of CASP3, leading to premature labor. Thus, it is the balance between uterine stress-mediated activation of non-apoptotic CASP3 and augmented adaptive UPR signaling that facilitates quiescence. Therefore, we hypothesize that normal transient stress insults may precondition the uterus and prevent contractility by increasing the capacity of the myometrium to experience ER stress in the absence of precocious increases in adaptive UPR signaling. Thereby maintaining tocolytic non-apoptotic CASP3 action and preventing premature labor.

To examine the role of endogenous pregnancy-dependent preconditioning on the maintenance of UPR-dependent non-apoptotic CASP3 activity, we initially preconditioned the UPR of the telomerase immortalized human uterine myocyte cell line (hTERT-HM), which has previously been demonstrated to be comparable to primary myocyte cultures with functional equivalency and similar expression of α smooth muscle actin, oxytocin receptor, estrogen receptor α, calponin, and caldesmon^17^, utilizing minor concentrations of tunicamycin (TM) and thapsigargin (Thaps), prior to the exposure of a known cytotoxic dose. These studies clearly demonstrated in vitro UPR preconditioning facilitates improved uterine myocyte cell viability preventing apoptotic consequences in the presence of elevated levels of CASP3 activation. In vivo, we utilized a novel pregnant mouse model where downstream stress-mediated UPR preconditioning effects were ablated by heightening tolerance to the gestational stresses through the administration of the chemical chaperone phenyl butyric acid (PBA). Interestingly, we observed increased apoptotic CASP3 activity within the endometrium, which lead to augmented prostanoid signaling, resulting in the onset of preterm birth in over 50% of the stressed-sub-preconditioned mice. Whereas endogenously preconditioned mice exposed to the same exogenous stress had a heighted capacity to maintain non-apoptotic CASP3-mediated quiescence, as ~83% delivered at term.

## Results

### UPR preconditioning renders CASP3 non-apoptotic in human uterine myocytes

Prior to commencing this study, we first tested the effects of minor concentrations of stress (TM) on the activation of prosurvival, UPR and inflammatory signaling responses for the potential use as a preconditioning stimulus (Supplementary Figure S1). In the context of preconditioning, the ideal stress stimulus should capacitate prosurvival signaling, i.e., GRP78, without inducing pro-apoptotic responses. We observed 24 h of 0.1 μg/ml TM significantly induced the GRP78 expression, in the absence of apoptosis or inflammation (Supplementary Figure S1). Specifically, the preconditioning stimulus did not increase activating transcription factor 4 (ATF4), nuclear factor kappa B (NF-κB), active CASP3, or GADD153 expression.

**Fig. 1:**
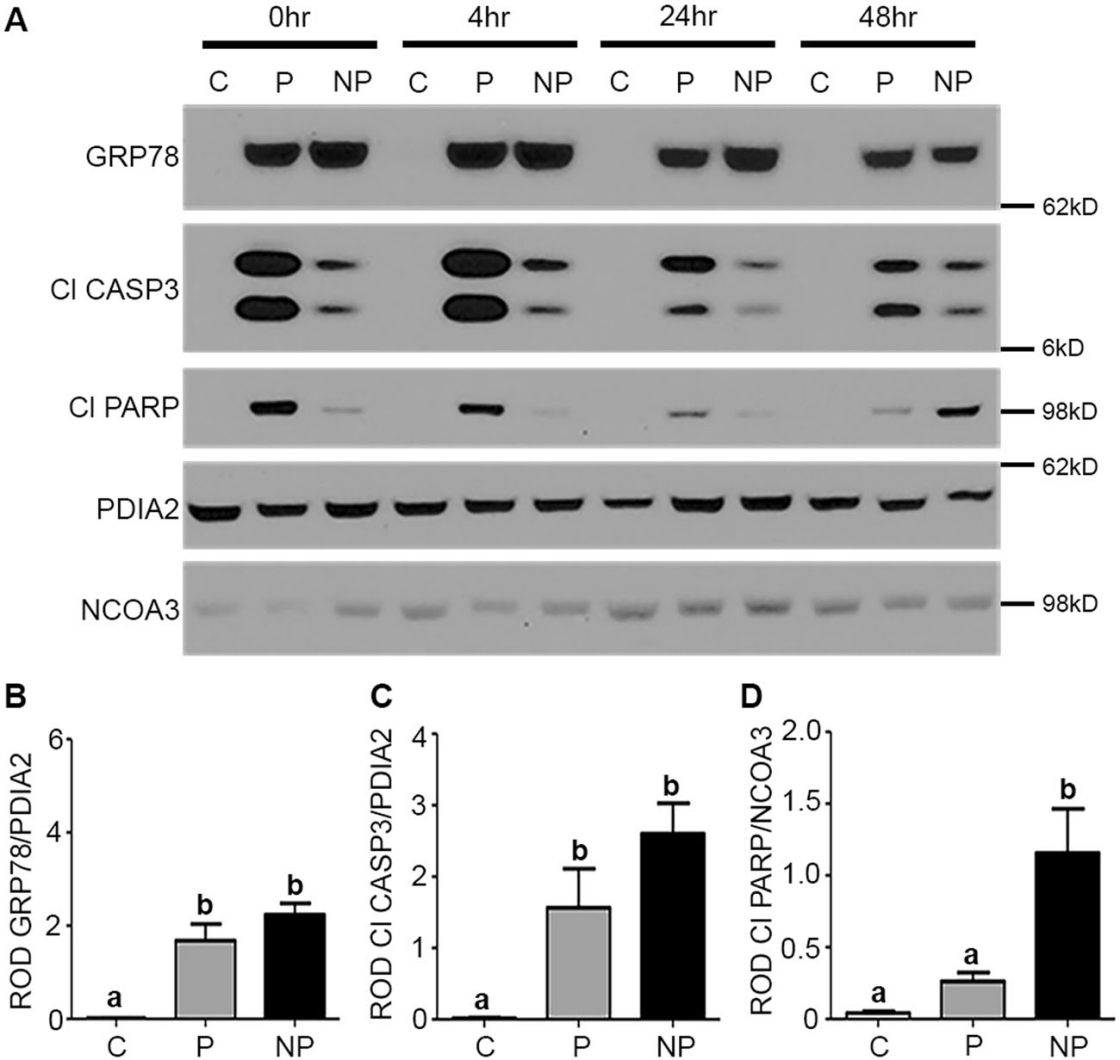
UPR preconditioning renders the hTERT-HM uterine myocyte CASP3 non-apoptotic. **a** Elevated levels of cytoplasmic GRP78 and Cl CASP3, and nuclear Cl PARP are observed in preconditioned (P) and non-preconditioned (NP) uterine myocytes as compared to controls (C) (*n* = 3 per condition), when exposed to a cytotoxic dose of TM 0, 4, 24, and 48 h post TM preconditioning. At 48 h to recovery there is equal activation of GRP78 (**b**) and Cl CASP3 (**c**) in both P and NP uterine myocytes. In contrast, Cl PARP (**d**) is significantly decreased in the P versus NP cells. PDIA2 and NCOA3 are utilized as cytoplasmic and nuclear protein loading controls. A representative blot from this experiment is shown. Statistical comparisons were performed using one-way ANOVA, and subsequent Newman–Keuls multiple-comparison tests. Data labeled with different letters are significantly different from each other (*p* < 0.05)

Subsequently, activation of the UPR, CASP3, and apoptotic indices were next examined by immunoblotting in control, preconditioned (0.1 μg/ml, 24 h TM) and non-preconditioned (vehicle) hTERT-HM cells, given a 0, 4, 24 and 48 h recovery period prior to a subsequent known cytotoxic dose of TM (5.0 μg/ml, 1 h) (Fig. 1)^18^. A robust activation of the UPR was observed in the levels of GRP78 and CASP3 in both the TM preconditioned and non-preconditioned cells (NP) compared to the vehicle control (Fig. 1a–c). Examination of apoptotic indices, as quantified by cleavage of the nuclear DNA repair molecule PARP, demonstrated that at all recovery time points examined CASP3 and PARP cleavage levels were equivalent, except at the 48 h recovery time point. With 48 h to recover, the uterine myocytes of the preconditioned and NP displayed equal levels of CASP3 cleavage. However remarkably the preconditioned cells had a 4-fold decrease in PARP cleavage compared to NP, demonstrating an acquired resistance to the apoptotic consequences of CASP3 activation (Fig. 1d). Decreased cell viability of the NP in comparison to control (C) and preconditioned (P) myocytes was further validated using a trypan blue assay (Supplementary Figure S2).

To validate that the observed anti-apoptotic effects of preconditioning were not modality-dependent, we repeated our preconditioning protocol using Thaps. In which case, activation of the UPR, CASP3, and apoptotic indices were next examined by immunoblotting in control, preconditioned (10 nM, 24 h Thaps) and non-preconditioned (vehicle) hTERT-HM cells, given a 48 h recovery period prior to a subsequent known cytotoxic dose of Thaps (250 nM, 1 h). Like TM preconditioning, activation of GRP78 was similar within the Thaps preconditioned (P) and non-preconditioned (NP) hTERT-HM cells compared to controls (C) (Supplementary Figure S3A and B). Importantly, in a manner similar to the TM preconditioning protocol Thaps-preconditioning reduced PARP cleavage by 2-fold (Supplementary Figure S3A – D) in the presence of a 25-fold increase in CASP3 activation (Supplementary Figure S3A – C) post preconditioning.

### UPR preconditioning inhibits inflammation in the human uterine myocyte

**Fig. 2:**
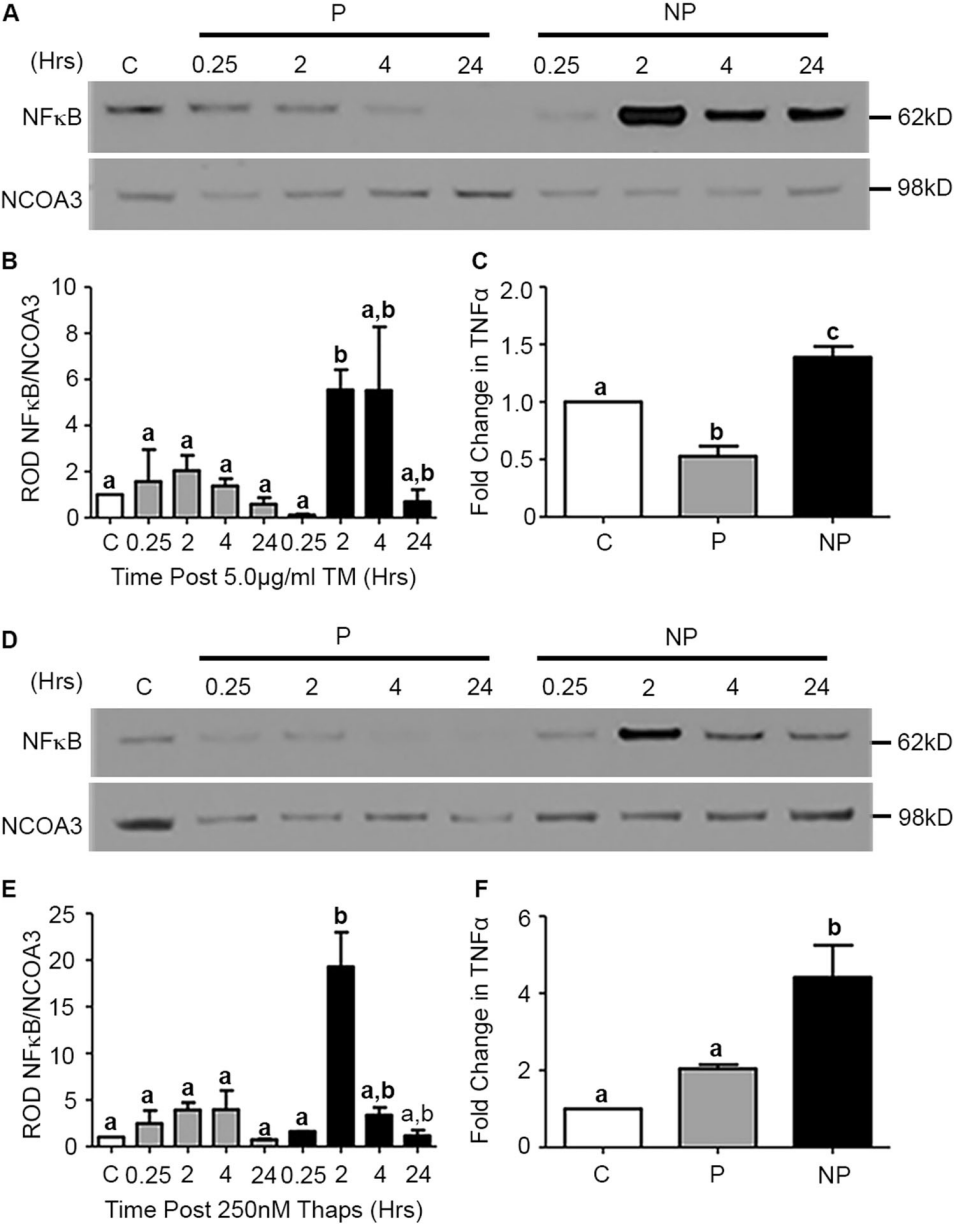
UPR preconditioning ablates NF-κB activation in the hTERT-HM uterine myocyte. **a**, **b**, **d**, **e** Activation of NF-κB was significantly increased in both TM and Thaps non-preconditioned (NP) cells and reduced to barely detectable levels in preconditioned (P) cells 2 h post administration of a cytotoxic dose of TM/Thaps. **c**, **f** TNFα secretion was also reduced in P versus NP cells. A representative blot from each experiment is shown. NCOA3 is utilized as nuclear protein loading control. Statistical comparisons were performed using one-way ANOVA, and subsequent Newman–Keuls multiple-comparison tests. Data labeled with different letters are significantly different from each other (*p* < 0.05)

To define the mediators facilitating resistance to the apoptotic consequences of CASP3, NF-κB activation in the nuclear compartment of the uterine myocyte was examined in control, preconditioned and NP exposed to TM, Thaps, or vehicle treatment. Cells were collected 0.25, 2, 4, and 24 h post administration of the cytotoxic bolus and compared to vehicle-treated controls. As seen in Fig. 2a, b, NP display a robust 5.5-fold activation of NF-κB 2 h post administration of the cytotoxic bolus, whereas NF-κB activation remains barely detectable in preconditioned cells at all time points examined post bolus (0.25, 2, 4, 24 h).

Enzyme-linked immunosorbent assays (ELISA) performed on control preconditioned and NP hTERT-HM cells collected 48 h post TM bolus revealed TNFα secretion was suppressed 0.5-fold in the preconditioned cells whereas, NP (Fig. 2c) demonstrated a 0.5-fold increase in levels compared to non-treated controls. Similar results were found when cells were preconditioned and stressed with Thaps (Fig. 2d). NF-κB activation and TNFα secretion was increased 19-fold 2 h post and 4-fold 48 h post exposure to the cytotoxic bolus (Fig. 2e, f) respectively, in NP but continued to remain suppressed in preconditioned cells.

### Preconditioning downregulates UPR activated apoptotic signaling

**Fig. 3:**
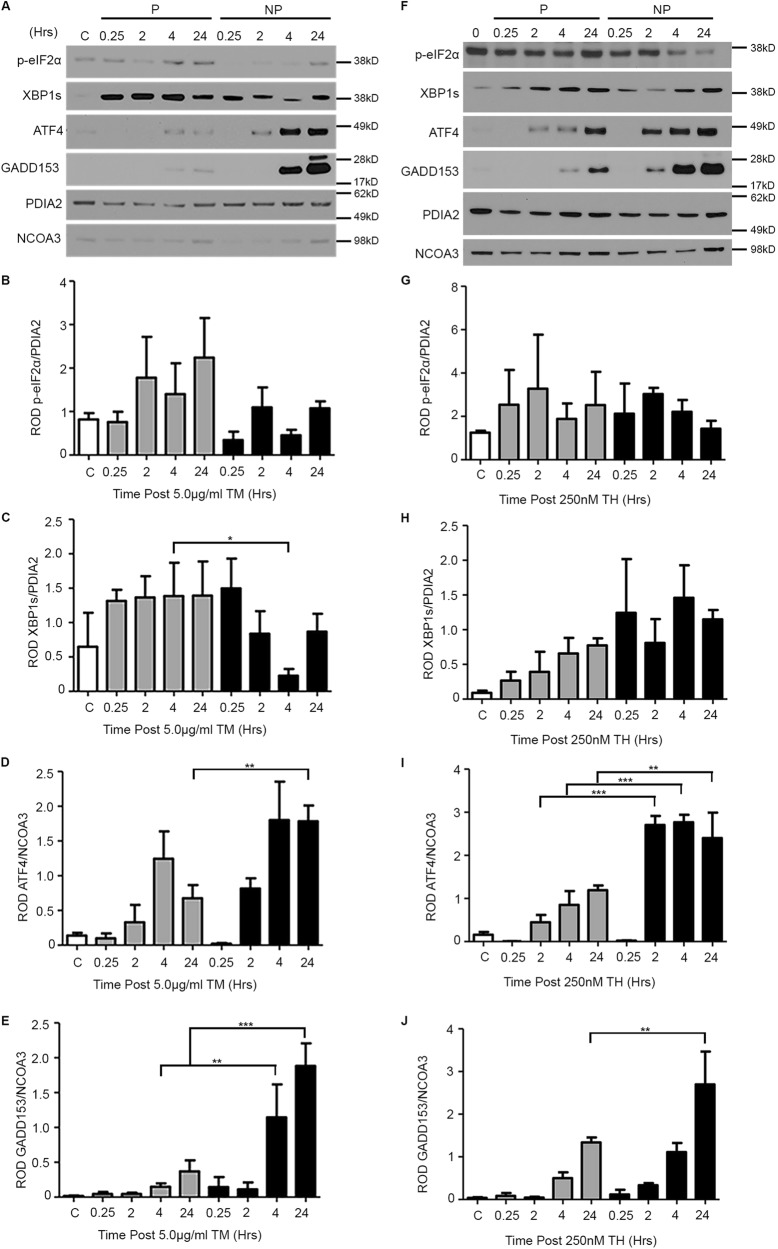
UPR preconditioning differentially regulates activation of the pro and anti-apoptotic arms of the UPR in the hTERT-HM uterine myocyte. TM (**a**) or Thaps (**f**) mediated preconditioning blocked activation of the pro-apoptotic arms of the UPR with ATF4 (**d**, **i**) and GADD153 (**e**, **j**) and TM preconditioning maintained activation of the anti-apoptotic arm of the UPR with XBP1s (**c**) significantly upregulated in preconditioned (P) versus non-preconditioned (NP) cells post administration of a cytotoxic dose of TM/Thaps. No changes in XBP1s (**h**) upon Thaps treatment, and p-eIF2α (**b**, **g**) upon TM and Thaps treatment. PDIA2 and NCOA3 are utilized as cytoplasmic and nuclear protein loading controls. A representative blot from each experiment is shown. Statistical comparisons were performed using one-way ANOVA, and subsequent Newman–Keuls multiple-comparison tests. *p < 0.05, **p < 0.01, and ***p < 0.001 compared with controls

We quantified stress-mediated activation of the UPR prosurvival (spliced XBP1 (XBP1s) and phospho-elongation initiation factor 2α (p-eIF2α)) and pro-apoptotic (ATF4 and GADD153 signaling pathways by immunoblotting immediately following the application of the TM or Thaps bolus (0.25, 2, 4, or 24 h post bolus) in control, preconditioned, and non-preconditioned hTERT-HM cells (Fig. 3a, f). p-eIF2 α levels remain unchanged between preconditioned and NP, TM, or Thaps (Fig. 3b, g). XBP1s levels were suppressed 4 h post bolus in TM-preconditioned cells (Fig. 3c), whereas no change in expression is observed between Thaps-preconditioned and NP (Fig. 3h). Pro-apoptotic signaling pathways in contrast, were significantly downregulated in both TM and Thaps preconditioned versus NP. A 2-fold decrease in ATF4 at 24 h (Fig. 3d) and a 7- and 5-fold reduction of GADD153 at 4 and 24 h, respectively (Fig. 3e) was observed in TM-preconditioned cells. Similarly, a 0.5-fold reduction in ATF4 at 24 h (Fig. 3i) and a 2-fold reduction in GADD153 at 2, 4, and 24 h (Fig. 3j) were observed in Thaps-preconditioned compared to non-preconditioned hTERT-HM cells.

Anti-apoptotic factors such as XIAP, MCL1, and Survivin have previously been demonstrated to increase in the uterine compartment of the pregnant mouse across gestation^19^. Increased prosurvival signaling is thus thought to play a role in maintaining myometrial CASP3 in a non-apoptotic state throughout early and mid-gestation to prolong uterine quiescence. Thus we examined the effects of preconditioning on the expression of anti-apoptotic factors XIAP and MCL1. We observed that XIAP and MCL1 were preferentially maintained in TM-preconditioned cells (Supplementary Figure S4A, B and C). However, no significant differences were found in XIAP and MCL1 expression between Thaps-preconditioned, non-preconditioned, and control hTERT-HM cells (Supplementary Figure S4D, E and F).

### Sub-preconditioned stressed mice have an increased incidence of preterm

We examined the timing of labor following a minor stress (0.2 mg/kg TM) on gestation day 16 in a sub-preconditioned (TM + PBA) and an endogenously preconditioned (TM) population of timed pregnant mice (*n* = 7 and *n* = 6, respectively). The effects of PBA and vehicle (Con) alone were also examined (*n* = 3 for both groups). About 57% of the TM + PBA, mice (4/7) delivered preterm, with an average delivery time of 30 h post TM administration (Table 1). In contrast, the mice that experienced normal endogenous gestational stressors prior to the delivery of a minor stress (TM) had a preterm birth rate of 17% (Table 1). No effects were observed in the timing of birth from the mice administered PBA alone (3/3), similar to Con mice (3/3), which delivered at term on E19 (Table 1). All animals that delivered at term resulted in live pups.

**Table 1:** In vivo preconditioning prevents preterm birth

	Term birth	Preterm birth	Percent preterm
Control	3	0	0%
PBA	3	0	0%
TM+PBA	4	3	57%
TM	1	5	17%

**Fig. 4:**
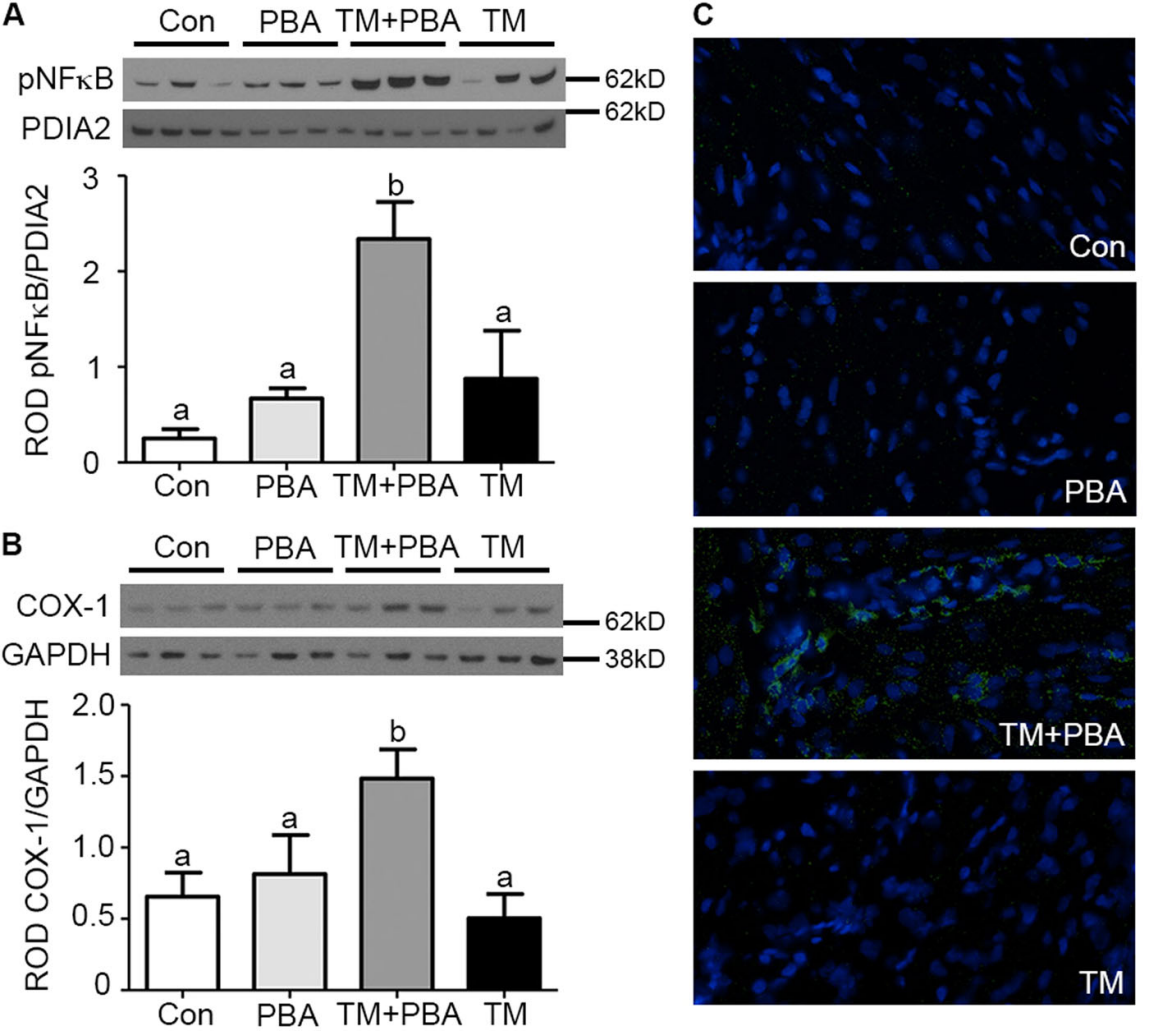
Endogenous preconditioning prevents premature activation of uterine inflammation in the pregnant mouse. Uteri collected from vehicle treated (Con), sub-preconditioned (PBA), exogenously stressed-sub-preconditioned (TM+PBA) and exogenously stressed-preconditioned (TM) mice on E17 prior to the onset of preterm or term birth were examined for **a** NF-κB, **b** COX-1, and **c** macrophage infiltration. Increased NF-κB activation, COX-1 expression, and elevated levels of macrophage infiltration were isolated to the TM+PBA uteri. PDIA2 and GAPDH are utilized as cytoplasmic loading controls. A representative blot or immunohistochemical image from each experiment is shown. Statistical comparisons were performed using one-way ANOVA, and subsequent Newman–Keuls multiple-comparison tests. Data labeled with different letters are significantly different from each other (*p* < 0.05)

We examined components of the inflammatory signaling cascade in the uteri collected from Con, PBA, TM + PBA, and TM treated pregnant mice at E17 prior to the onset of term and preterm birth. Uterine NF-κB activation, macrophage infiltration, COX-1, and COX-2 levels were examined (Fig. 4). Premature uterine activation of NF-κB occurs in the stressed-sub-preconditioned (TM+PBA) mice prior to the onset of labor, a 2.7-fold increase in p65 nuclear translocation was observed when compared to control animals (Fig. 4a). Immunohistochemistry validated the observed increased NF-κB activation in stressed-sub-preconditioned mice, and revealed the increased activity occurred within both the myometrial and endometrial compartments (Supplementary Figure S5A and B, respectively). While COX-2 levels remained undetectable, COX-1 levels significantly increased over 3-fold in the TM + PBA uteri (Fig. 4b) in comparison to Con uteri. Macrophage infiltration of the uterine tissue of the Con, PBA, TM+PBA and TM treated mice was examined by F4/80+ immunofluorescence analysis and a greater than 10 fold increase in the number of macrophages was observed in the TM + PBA uteri that consequently undergo preterm birth in comparison to the PBA, TM and Con uteri (Fig. 4c).

### Apoptotic CASP3 activity in the pregnant uterus increases contractility

**Fig. 5:**
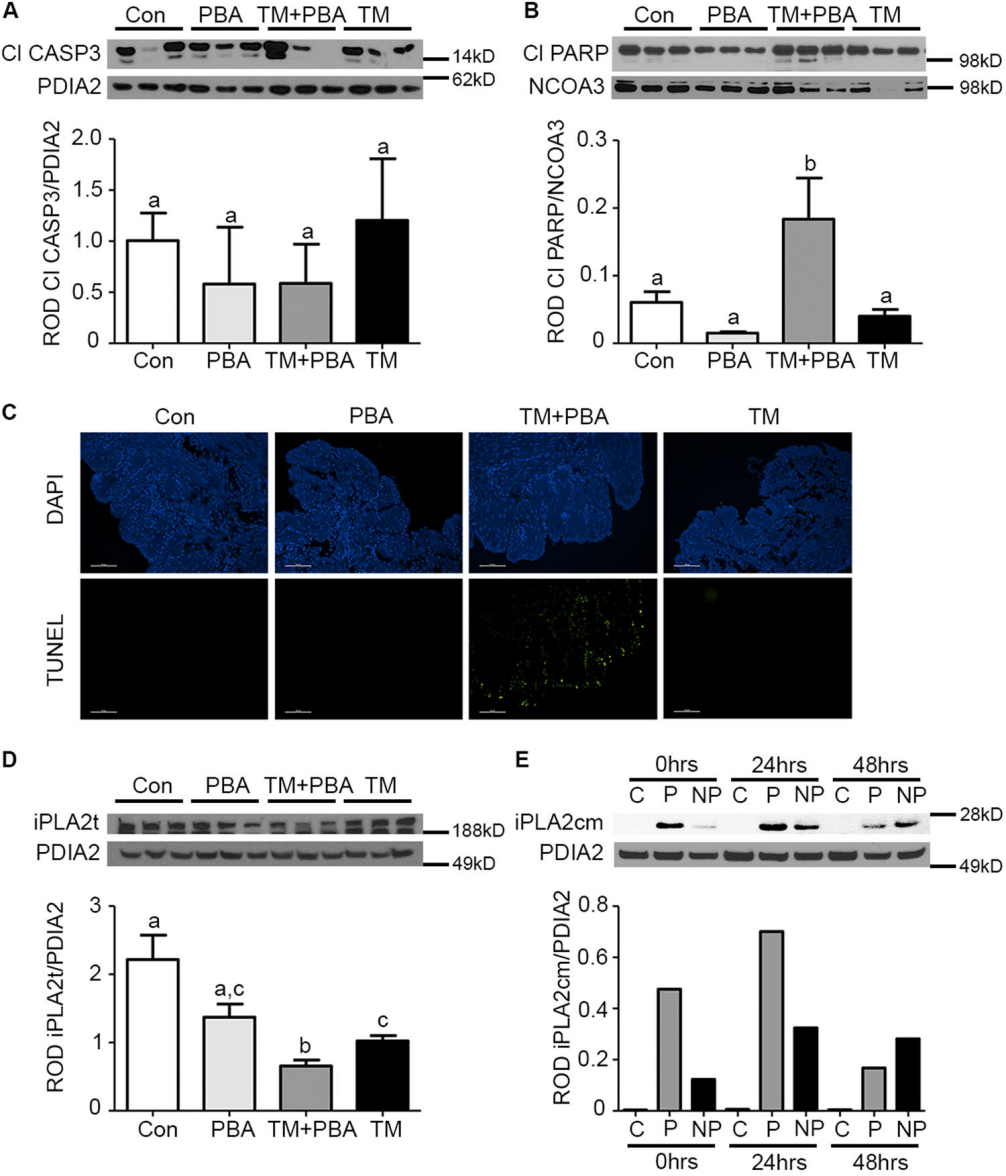
Endogenous preconditioning facilitates the maintenance of non-apoptotic CASP3 and suppresses iPLA2 activation in the pregnant mouse uterus. Uteri collected from vehicle treated (Con), sub-preconditioned (PBA), exogenously stressed-sub-preconditioned (TM+PBA) and exogenously stressed-preconditioned (TM) mice on E17 prior to the onset of preterm and term birth were examined for **a** active Cl CASP3. **b** Cl PARP, and **c** TUNEL staining to measure apoptotic cell death. iPLA2t levels act as an indirect measure of iPLA2 activation (**d**). Cl CASP3 levels remained unchanged across all 4 groups examined however increased Cl PARP and TUNEL activity and decreased levels of the inactive iPLA2t were isolated to the TM+PBA-treated mice. **e** In the hTERT-HM cells the cleaved active monomeric form of iPLA2 (iPLA2cm) was elevated in a relative manner to the levels of apoptotic CASP3 present in the preconditioned and non-preconditioned cells (Fig. 1a). A representative blot or image from each experiment is shown. PDIA2 and NCOA3 are utilized as cytoplasmic and nuclear protein loading controls. Statistical comparisons were performed using one-way ANOVA, and subsequent Newman–Keuls multiple-comparison tests. Data labeled with different letters are significantly different from each other (*p* < 0.05)

Uteri isolated from Con, PBA, TM + PBA, and TM mice were examined prior to the onset of term or preterm labor at E17 by immunoblotting for CASP3 activation. Levels of CASP3 activation were not significantly changed between the 4 groups examined (Fig. 5a). However, the TM + PBA uteri demonstrate increased incidence of apoptotic CASP3 activation as indicated by a 4.6-fold increase in the levels of uterine PARP cleavage (Fig. 5b) when compared to PBA, Con, or TM treated uteri. Positive terminal deoxynucleotidyl transferase dUTP nicked-end labeling (TUNEL) staining in the sub-preconditioned uteri, but not PBA, Con, or TM treated validated these results (Fig. 5c).

We observed a 2-fold decline in the homotetromeric non-active form of iPLA2 (Fig. 5d) in the apoptotic CASP3-positive (Fig. 5a, b) TM + PBA uteri in comparison to the Con and PBA-treated uteri. In the hTERT-HM we were able to detect the cleaved active form of iPLA2 and observed a 2-fold increase (Fig. 5e) isolated to the NP, which also display elevated levels of apoptotic CASP3 as indicated by the pattern of PARP cleavage in Fig. 1a.

**Fig. 6:**
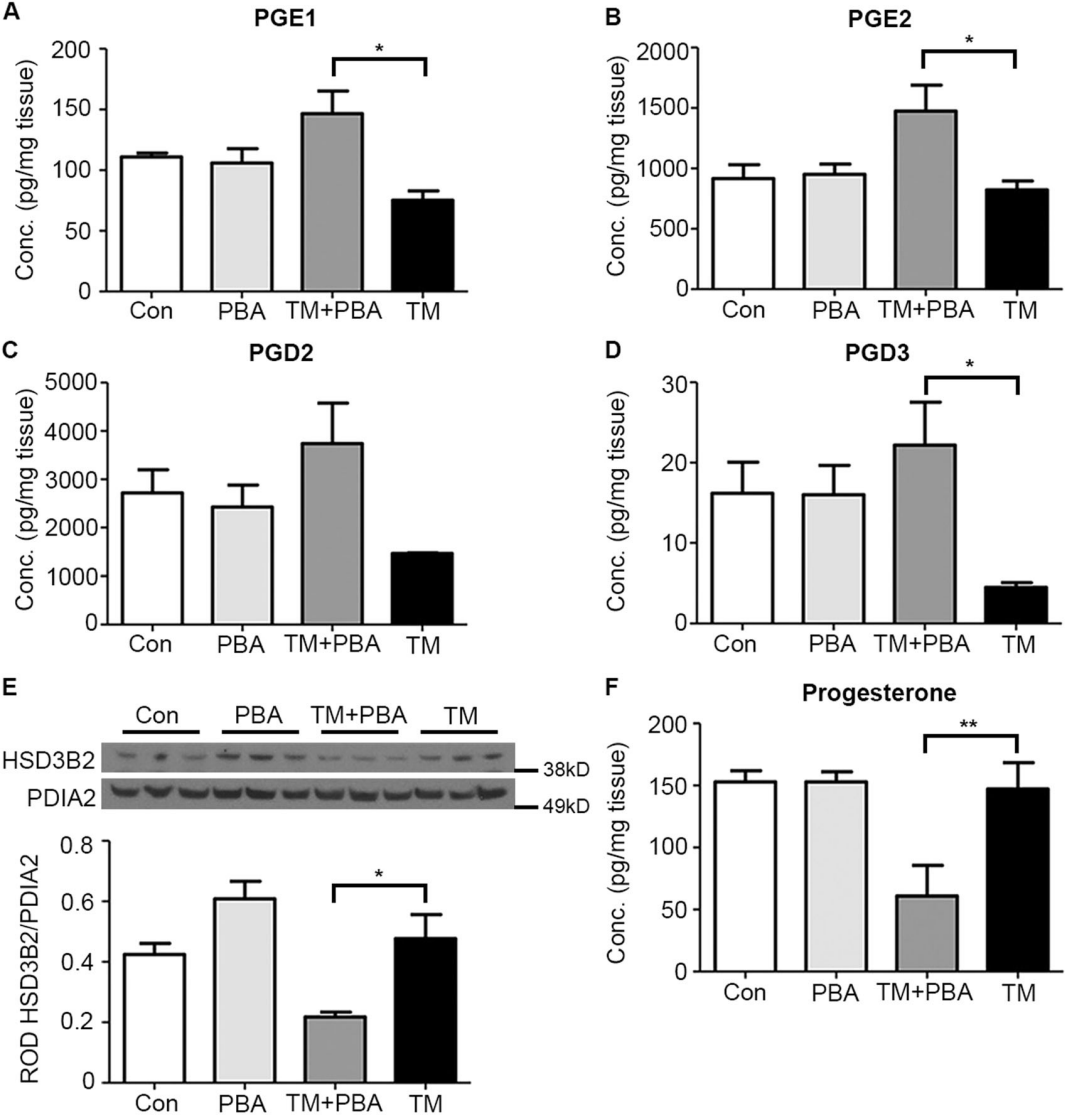
Preconditioning facilitates the suppression of prostaglandin synthesis thereby preventing premature luteolysis and P4 withdrawal. Uteri collected from vehicle treated (Con), sub-preconditioned (PBA), exogenously stressed-sub-preconditioned (TM+PBA) and exogenously stressed-preconditioned (TM) mice on E17 prior to the onset of preterm and term birth, were examined for prostaglandin production. Significantly elevated levels of **a** PGE1, **b** PGE2, and **d** PGD3 were isolated to TM+PBA uteri. **e** Ovarian HSD3B2 and **f** serum P4 levels were significantly decreased in the TM+PBA-treated mice. A representative blot from each experiment is shown. PDIA2 is utilized as cytoplasmic protein loading control. Statistical comparisons were performed using one-way ANOVA, subsequent Newman–Keuls multiple-comparison tests and Student's *t*-test. *p ≤ 0.05 and **p ≤ 0.01 compared with controls

Uteri isolated from Con, PBA, TM + PBA, and TM treated mice examined at E17 for prostaglandin production within the uterine compartment utilizing targeted-small molecule liquid chromatography and tandem mass spectrometry. Significantly elevated levels of PGE2, PGE1, and PGD3 were isolated to the sub-preconditioned mice exposed to a minor exogenous stress (TM+PBA) (Fig. 6a, b, d). Furthermore, downstream by-products of arachidonic acid metabolism, e.g., 11-HETE and 13-HODE, were also significantly elevated in sub-preconditioned mice compared to preconditioned controls (Supplementary Figure S6).

Ovaries were collected from Con, PBA, TM + PBA and TM treated pregnant mice on E17 and analyzed for 3β hydroxysteroid dehydrogenase (HSD3B2), which declines with the onset of luteolysis. As seen in Fig. 6e, there was a significant reduction (2.2-fold) in HSD3B2 expression in the ovaries of the TM+PBA mice in comparison to Con, PBA, and TM ovaries. To validate premature luteolysis in TM+PBA pregnant mice, serum collected on E17 from each cohort of mice was analyzed with ELISA for circulating progesterone levels. As observed in the TM+PBA mice that undergo preterm delivery (Table 1), there was a significant decline (2.4-fold) in circulating P4 levels in comparison to Con, PBA, and TM treated mice (Fig. 6f).

## Discussion

We have previously demonstrated the critical role the UPR plays in non-apoptotic CASP3 activation within the uterine compartment during pregnancy^1^. The current study is based on the findings that the act of preconditioning the uterine UPR during pregnancy is essential in protecting the pregnant uterine myocyte against a CASP3-mediated apoptotic fate. We also demonstrate that uterine preconditioning of the UPR during pregnancy promotes prolonged uterine myocyte quiescence through the suppression of apoptotic CASP3-mediated activation of two interdependent signaling pathways, the inflammatory and iPLA2-prostaglandin signaling cascade. Increased uterine inflammation and prostaglandin signaling herald the end of gestation and are normally associated with the onset of labor in both human and mouse^20^. Consequently, we propose that in the absence of appropriate preconditioning or an inability to host an appropriate UPR to preconditioning stimuli, the uterine compartment is placed at an increased risk for precocious apoptotic CASP3 activation, which results in a heightened incidence of preterm birth.

CASP3 activity is commonly associated with the initiation of cellular apoptosis^21^. However it has repeatedly been demonstrated that despite high levels of active CASP3, the pregnant myometrium avoids cellular apoptosis throughout the entirety of gestation and postpartum involution, whereas the endometrium displays increased apoptotic CASP3 activity at term and during the involution process^22,23^. While CASP3 targets were not identified in the smooth muscle of the bladder, CASP3 was found to target structure proteins in both the heart and diaphragm. In cardiac tissue specifically, both α-actin and α-actinin, components of the cardiac contractile architecture were directly cleaved by CASP3 and then further degraded^13–15^. Similarly, we have shown in the absence of apoptotic consequences non-apoptotic uterine CASP3 protease activity inhibits myometrial contraction in a tocolytic manner through the targeted cleavage and degradation of multiple components of the contractile architecture, i.e., connexin 43, α-actin, and γ-actin, limiting uterine myocyte contractile potential^1,19^.

Preconditioning refers to the phenomenon where sub-lethal insults can induce tolerance to subsequent stressors. The process of preconditioning biological systems against pathophysiological events can be observed in multiple forms. As an example, ischemia/reperfusion preconditioning has become an increasingly active area of interest^24–26^ and it has been demonstrated that multiple applications of brief ischemic events prior to prolonged ischemia significantly reduces subsequent tissue damage and limits the apoptotic outcomes associated with CASP3 activation^3–5^. Both in vivo and in vitro studies have proven that preconditioning acts through the endoplasmic reticulum with minor insults of stress stimulating a protective adaptive UPR promoting tolerance and anti-apoptotic signaling fostering resistance to the apoptotic processes associated with subsequent, more damaging stresses^18,27^. This preconditioning phenomenon is strongly conserved in evolution^28^ and can be induced by many and varied perturbations such as stress-mediated inflammation, hypoxia, and cytotoxins^18,29,30^. Prior to this study, data pertaining to the role of preconditioning the uterine UPR in the regulation of gestational length was limited. It was known however, that women who exercise during pregnancy have a reduced risk of preterm delivery^31^. Whereas exercise in non-pregnant women has been demonstrated to acutely activate the UPR to facilitate adaptation of skeletal muscle^32^. Exercising while pregnant may be acting in a preconditioning-like manner to increase myometrial tolerance to subsequent stressors. More relevantly, studies in nonhuman primates examining the effects of uterine overdistention on preterm labor have found significant evidence of uterine preconditioning^33^. Specifically, they show that additional stretch to the pregnant uterus at approximately the start of the third trimester via intraamniotic balloons lead to the onset of preterm labor. Yet, when the days to maximum inflation of the balloons were extended from 1 day to 2 days, preterm labor did not occur and all nonhuman primates delivered at term. Importantly, the maximum volume or stretch induced did not differ between the two experiments. While these studies did not delve into the mechanism of sustained quiescence they do suggest the initial stretch stimulus to the pregnant uterus facilitated myometrial adaption preventing preterm labor in response to the subsequent stretch. Our in vitro data reveal preconditioning of the uterine myocyte UPR, suppressed inflammatory responses (Fig. 2), enhanced prosurvival signaling (Fig. 3), and promoted non-apoptotic CASP3 activation (Fig. 1) during periods of cellular stress. As can be observed in Fig. 1, despite equally elevated levels of CASP3 activation (Fig. 1c) in preconditioned and NP 48 h post receiving a bolus (Fig. 1), the preconditioned cells demonstrated a newly acquired resistance to CASP3-mediated apoptotic cell death as indicated by decreased PARP cleavage (Fig. 1d). Suppressed activation of NF-κB and consequently decreased secretion of TNFα (Fig. 2c) were also observed upon uterine myocyte UPR preconditioning (Fig. 2). Furthermore preconditioning of the UPR promotes inhibition of the pro-apoptotic arms and enhanced activation of the protective arms of the UPR, respectively (Fig. 3, Supplementary Figure S1). Taken together these data demonstrate that preconditioning of the uterine myocyte plays a critical role in maintaining the uterine myocyte in a CASP3-positive, non-apoptotic, anti-inflammatory, prosurvival phenotype in vitro. Our in vivo analysis of the stressed-preconditioned and stressed-sub-preconditioned pregnant mouse uteri established that the preconditioned non-apoptotic phenotype is critical for the maintenance of an appropriate gestational length through the preservation of uterine quiescence. In vivo analysis of a stressed-sub-preconditioned pregnant mouse model (TM+PBA) revealed they were more susceptible to a preterm birth phenotype (57%) than mice that experienced normal endogenous preconditioning prior to the delivery of a minor exogenous stress (TM), which displayed a preterm birth rate of only 17% (Table 1). As can be observed in Fig. 4a, the uterus of the TM+PBA mice also demonstrated significantly elevated levels of macrophage infiltration, NF-κB activation, and COX-1 expression (Fig. 4a-c). These data demonstrate that appropriate endogenous preconditioning maintains the uterine capacity to suppress inflammatory signaling cascades late in gestation within the uterine compartment protecting against subsequent exogenous stressors. Moreover, while uterine CASP3 levels remained unmodified across treatments (Fig. 5a), the stressed-sub-preconditioned mice (TM+PBA) displayed increased uterine apoptotic CASP3 as indicated by both increased PARP cleavage (Fig. 5b) and elevated TUNEL staining observed primarily within the endometrial compartment (Fig. 5c). These data demonstrate that preconditioning plays a vital role in protecting the endogenously stressed uterine compartment from undergoing a precocious apoptotic CASP3-mediated cell death.

It has previously been established that CASP3 activity is a critical upstream component in prostaglandin production through cleavage and activation of iPLA2 allowing the release of free arachidonic acid to be converted into prostaglandins in a COX1/2- and NF-κB-dependent manner^34,35^. However, this study reveals that it must be apoptotic CASP3 activity for the appropriate initiation of prostaglandin synthesis while non-apoptotic CASP3 though active is unable to do so. Upon apoptotic CASP3 activation (TM+PBA), we observed decreased levels of the inactive uterine iPLA2 in vivo (Fig. 5c, d). In vitro the cleaved active form of iPLA2 was readily detectable (Fig. 5e) and found to be significantly upregulated in the presence of apoptotic CASP3 and reduced in the presence of non-apoptotic CASP3 (48 h P versus NP Fig. 1a). iPLA2 activation in response in precocious apoptotic CASP3 in the uterine compartment of the TM+PBA mouse resulted in precocious prostaglandin production (Fig. 6), triggering luteolysis as evidenced by decreased ovarian HSD3B2 (Fig. 6e)^36^ and consequently decreased circulating P4 levels (Fig. 6f) and the onset of preterm birth limited to the TM+PBA-treated mice (Table 1).

Taken together, these data demonstrate for the first time that uterine preconditioning, acting to suppress uterine apoptotic CASP3 activation and inflammation is placed upstream of regulating the timing of normal parturition by actively preventing the well-established endogenous uterotonic signaling cascades such as prostaglandin synthesis, luteolysis, and consequently P4 withdrawal that herald the onset of normal term labor in the pregnant mouse.

## Materials and methods

### Animals

The Institutional Animal Care and Use Committee of Wayne State University approved all animal studies. Timed pregnant female CD-1 mice (6–8 wks; gestation day 9) (Charles River Laboratories, Wilmington, MA) were housed in AALAC-accredited facilities according to IACUC guidelines. Accordingly, mice were given a standard pellet diet and water ad libitum.

### Cell culture

For the in vitro cell culture model system, we utilized telomerase immortalized human myometrial cells (hTERT-HM).^17^ In detail, human myometrial cells were collected from the anterior wall of the uterine fundus in women of reproductive age undergoing a hysterectomy. The catalytic subunit of telomerase was then expressed in the myometrial cells via retroviral infection. In these studies, hTERT-HM cells were cultured in Dulbecco-modified Eagle/F12 low glucose media (DMEM-F12) (Invitrogen Carlsbad, CA), supplemented with 10% fetal bovine serum (vol/vol) (Invitrogen) and antibiotic/antimycotic (10,000 U/ml; Invitrogen), and incubated at 37 °C with 95% air and 5% CO_2_.

### TM and thaps treatments in vitro

TM was suspended in 20 μl 10 M sodium hydroxide and brought to a final concentration of either 0.1 μg/ml or 1.0 μg/ml in DMEM-12 media with 10% FBS and antibiotic/antimycotic. Thaps (Sigma-Aldrich, St. Louis, MO; Cat#T9033) was dissolved directly in cell culture media and brought to a final concentration of 10 nM or 250 nM. For TM preconditioned (P) and non-preconditioned (NP) treatments hTERT-HM were given a 24 h treatment of 0.1 μg/ml TM or vehicle, respectively, 0–48 h prior to a secondary treatment of 5.0 μg/ml TM. Similarly, for Thaps preconditioned (P) and non-preconditioned (NP) treatments hTERT-HM cells were given a 24 h treatment of 10 nM TH or vehicle, respectively, 48 h prior to a secondary treatment of 250 nM Thaps. In both conditions, media were replaced 1 h after the secondary treatment was given and cells and media were collected 47 h later.

### TM and PBA treatments in vivo

PBA was directly dissolved into phosphate buffered saline (PBS) at pH 8.0 (Santa Cruz Biotechnology, Dallas, TX; sc-200652). TM (Calbiochem, San Diego, CA; Cat#654380) was initially dissolved in 20 μl 10 M sodium hydroxide and then suspended in PBS, pH 8.0. Sub-preconditioned pregnant CD-1 female mice (E10-15) were administered twice-daily intraperitoneal injections (i.p) of 50 mg/kg PBA, while preconditioned controls were administered PBS. At E16, stressed mice were administered 0.2 mg/kg TM i.p, while controls were given volume matched PBS. Following TM injections, the length of gestation was then monitored and compared between a subset of sub-preconditioned and endogenously preconditioned mice. Uteri, ovaries, and serum were harvested at E17 in the additional mice.

### Cytosol and nuclear protein fractionation

Cytoplasmic and nuclear protein fractions from hTERT-HM cells and frozen mouse tissues were prepared as previously mentioned. Initially, cells were rinsed in ice-cold PBS, centrifuged at 956× *g*, re-suspended, and evenly homogenized in ice-cold NE1 buffer (10 mM Hepes pH 7.5, 10 mM MgCl_2_, 5 mM KCl, 0.1% Triton X-100 with 1X EDTA-free protease/phosphatase inhibitor mini tablet). While tissues, were pulverized in liquid nitrogen and homogenized in ice-cold NE1 buffer. The homogenates were then centrifuged at 2655× *g*, the supernatant was retained as the cytoplasmic protein fraction and the pellet was washed in NE1 buffer and suspended in ice-cold NE2 buffer (20 mM Hepes pH 7.9, 500 mM NaCl, 1.5 mM MgCl_2_, 0.2 mM EDTA pH 8.0, 25% (vol/vol) glycerol with 1X EDTA-free protease/phosphatase inhibitor mini tablet). The homogenate was vortexed for 30 s every 5 min and after 1 h, centrifuged at 10,621× *g*. The supernatant was then retained as the nuclear fraction. Protein estimation was performed using a bicinchoninic acid (BCA) assay, equal amounts of protein were loaded for immunoblotting, PDI and NCOA3 were utilized as loading controls for the cytoplasmic and nuclear fractions, respectively.

### Immunoblotting and densitometric analysis

Equal amounts of protein were separated via electrophoresis on NuPAGE 4-12% gradient precast polyacrylamide gels (Life Technologies, Carlsbad, CA). Proteins were transferred onto Hybond-P PVDF membranes (Millipore, Billerica, MA) and blocked for 1 h at room temperature in 5% non-fat milk prepared in Tris Buffered Saline with 0.1%Tween-20 (vol/vol). Membranes were incubated with primary antibodies overnight at 4 °C. Primary antibody concentrations were as follows: GRP78 (1:1000; Cat#3177), Cl CASP3 (1:250; Cat#9664), GADD153 (1:500; Cat#5554), Cl PARP (1:1000; Cat#9541), ATF4 (1:500; Cat#11815), p-eIF2α (1:500; Cat#3398), NF-κB (1:1000; Cat#8242), XIAP (1:250; Cat#2042), and PDI (1:5000; Cat#3501) were obtained from Cell Signaling Technologies; XBP1s (1:500; Cat#37152) was obtained from Abcam; ATF6 (1:500; Cat#24169-1-AP) was obtained from Proteintech; MCL1 (1:1000; Cat#sc-819) was obtained from Santa Cruz Biotechnology; and NCOA3 (1:5000; Cat#PA1-845) was obtained from ThermoScientific. Following primary incubation, immunoreactivity was detected using horseradish peroxidase-conjugated secondary antibodies and visualized using an enhanced-chemiluminescence detection system (ThermoScientific, Rockford, IL). Immunoreactive band density was then quantified using ImageJ software.

### Enzyme-linked immunosorbent assay (ELISA)

In vitro, media samples were loaded into Amicon Ultra Centrifugal Filters (Millipore, cat#UFC500396) and centrifuged for 30 min at 14,000× *g* to concentrate media ~10×. The level of human tumor necrosis factor-alpha (TNFα) was then measured in 10X concentrated media using an ELISA. Specifically, the MSD Multi-Spot TNFα ELISA (Meso Scale Diagnostics, Rockville, MD, Cat#K151QWD) was performed according to the manufacturer’s instructions and results were read via the Meso Scale Discovery 1300 microplate reader. Each sample measurement was read in duplicate and the computed averages were taken based on the calculated standard curve.

In vivo, the level of progesterone (P4) was then measured in pregnant mouse serum using an ELISA. Specifically, the P4 ELISA Kit (Alpha Diagnostic International, San Antonio, TX, Cat#1955) was performed according to the manufacturer’s instructions and results were read via the Molecular Devices, SpectraMax M2 microplate reader. Each sample measurement was read in duplicate and the computed averages were taken based on the calculated standard curve.

### Terminal deoxynucleotidyl transferase dUTP nicked-end labeling assay

Tissues collected at E17, imbedded in optimal cutting temperature compound (Sakura Finetek USA Inc, Torrance, CA) were sectioned (10-μm thick), mounted onto Superfrost Plus Micro Slides, and stored at −20 °C. Sections were removed from storage and fixed in 4% paraformaldehyde for 15 min. Additionally, sectioned paraffin wax imbedded tissues were de-paraffinized and rehydrated and treated with 10 μg/ml Proteinase K for 15 min at 37 °C. Analysis of apoptosis in all tissues was quantified using the In Situ Cell Death Detection Kit, AP (Roche, Indianapolis, IN, Cat#11684809910) according to the manufacturer’s instructions.

### Small molecule liquid chromatography mass spectrometry analysis

Dissected uterine tissues separated into endometrial and myometrial compartments, flash frozen in liquid nitrogen, were removed from −80 °C storage and tissue weights were immediately recorded. Samples were then suspended in 1 ml cold PBS pH 7.4, homogenized via bead homogenization, and centrifuged at 10,621× *g* for 10 min. Supernatants were removed, and protein concentrations were determined using a BCA assay. Equal volume of protein (850 μl) was then spiked with 5 ng of internal standards suspended in 15% methanol dissolved in water (150 μl), mixed thoroughly, and purified using a C18 solid-phase cartridges. Prior to applying the sample, the cartridges were first washed with 1 ml of 100% methanol followed by 1 ml of 15% methanol. After the addition of the sample, tubes were rinsed twice with 1 ml of PBS and the rinse was passed through the cartridges. Subsequently, the cartridges were rinsed with 2 ml of hexane, vacuum dried for 30 s and proteins were eluted with 1 ml of methanol containing 0.1% formic acid. All samples were evaporated to dryness with a gentle stream of nitrogen at 40 °C, residues were re-suspended in 30 μl methanol and stored at −20 °C until LC-MS analysis. Prior to analysis, each sample was further diluted with 30 μl 25 mM aqueous ammonium acetate. Specific methods utilized for liquid chromatography mass spectrometry can be referenced in Yoon Park et al. 2014.

### Immunofluorescence

Tissues collected at E17, embedded in optimal cutting temperature compound (OCT) (Sakura Finetek USA Inc, Torrance, CA) were sectioned (10 μm thick), mounted onto Superfrost Plus Micro Slides, and stored at −20 °C. Sections removed from storage were fixed in 4% paraformaldehyde for 2 min. Fixed sections were incubated with primary antibody overnight at 4 °C and examined for primary immunoreactivity using a conjugated secondary antibody. The primary and secondary antibody concentrations were as follows: F4/80 + (1:250, Abcam, Cat#ab6640) diluted in PBS and detected by secondary goat anti-rat antibody conjugated to Alexa Fluor 488 (1:500, Abcam, Cat#150157), NF-κB (1:400, Cell Signaling Technologies, Cat#8242) diluted in PBS and detected by secondary donkey anti-rabbit antibody conjugated to Cy3 (1:500, Jackson Immunoresearch, Cat#711-165-152).

### Statistical analysis

All data represent at least three individual experiments performed in triplicate. For the direct comparison of three or more conditions a one-way analysis of variance was performed, with multiple comparisons analyzed via Newmans–Keuls multiple comparisons test. When directly comparing two conditions a two-tailed Student's *t*-test was performed. All comparisons were considered significant with *p*-values less than 0.05.

## Electronic supplementary material

Supplimental Information
