# Supplementary material for: Preconditioning the uterine unfolded protein response maintains non-apoptotic Caspase 3-dependent quiescence during pregnancy
Source: Cell Death Dis. 2018 Sep 17;9(10):933. doi: 10.1038/s41419-018-1000-4 (PMC6141493; doi:10.1038/s41419-018-1000-4)
Supplement: Supplementary file 1 — Supplimental Information [file 41419_2018_1000_MOESM1_ESM.docx]

**SFigure 1**


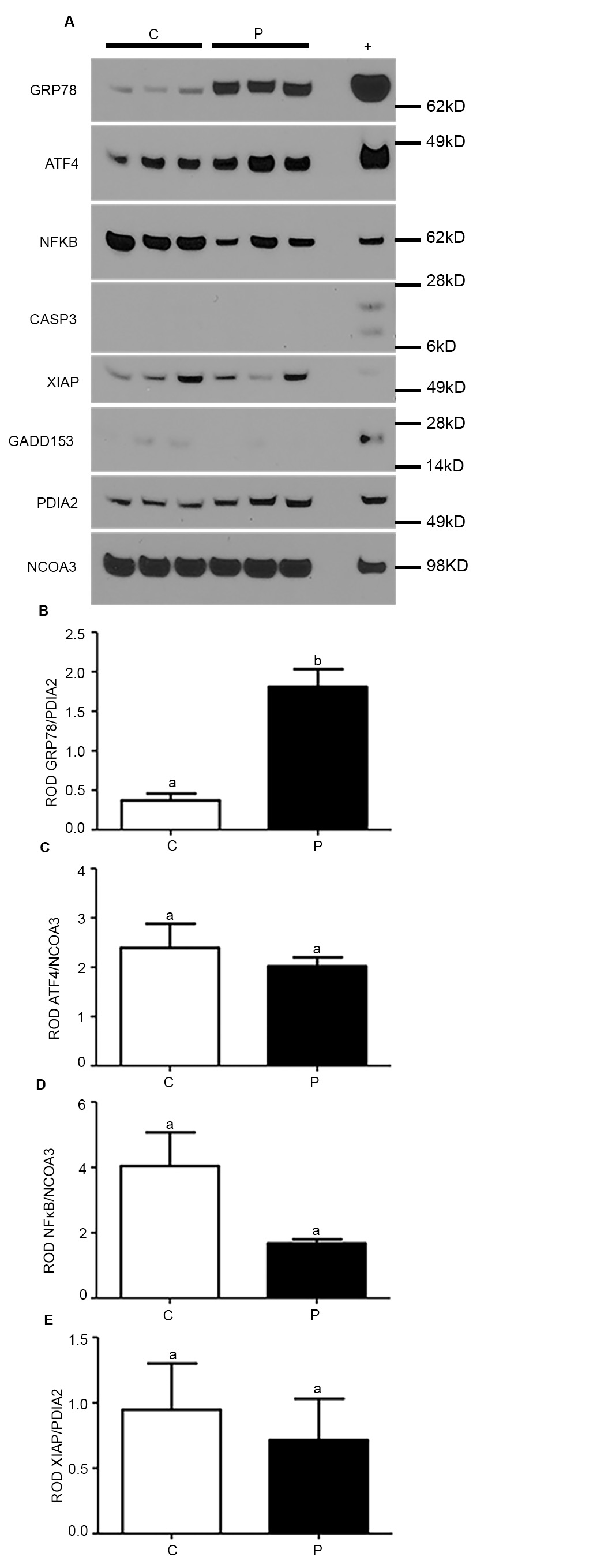


**Fig. S1.** Preconditioning dose tunicamycin has negligible impact on UPR, inflammatory, pro-apoptotic indices in the hTERT-HM uterine myocyte. GRP78, ATF4, NFκB, CASP3, XIAP, and GADD135 levels were measured in vehicle treated *(C)* uterine myocytes and preconditioned *(P)* myocytes exposed to a minor UPR stress (TM, 0.1$\mu$g/ml, 24hrs). GRP78 levels were modified significantly, whereas all others remained unchanged, indicating the lack of downstream consequences of the preconditioning stress alone *in vitro*. A representative blot from each experiment is shown. PDIA2 and NCOA3 are utilized as our cytoplasmic and nuclear protein loading controls. Statistical comparisons were performed using one-way ANOVA, and subsequent Newman-Keuls multiple-comparison tests. Data labeled with different letters are significantly different from each other (p<0.05).

**SFigure 2**


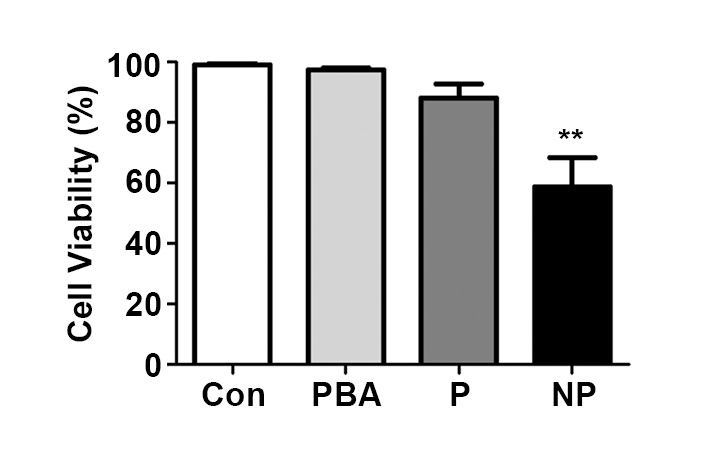


**Fig. S2.** UPR preconditioning increases cell viability of the hTERT-HM uterine myocyte in the presence of active non-apoptotic CASP3. Decreased cell viability was observed in non-preconditioned *(NP)* uterine myocytes as compared to controls *(C)* and preconditioned myocytes *(P)* (n=3 per condition), when exposed to a cytotoxic dose of TM 48 hrs post TM preconditioning as measured using a trypan blue assay. Statistical comparisons were performed using a one-way ANOVA, and subsequent Newman-Keuls multiple-comparison tests. *p≤0.05 and **p≤0.01 compared with controls.

**
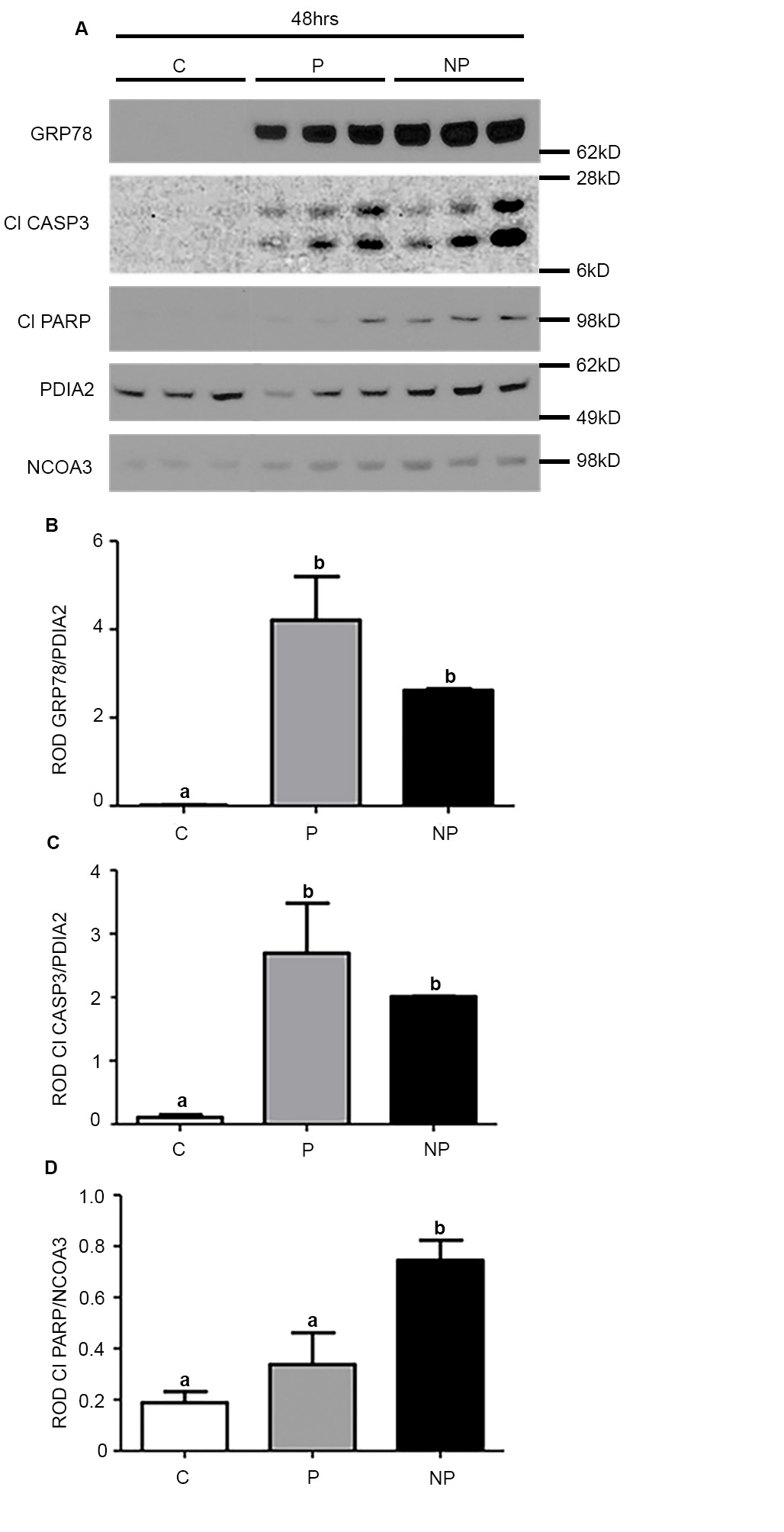
SFigure 3**

**Fig. S3.** Thapsigargin mediated UPR preconditioning renders the hTERT-HM uterine myocyte CASP3 non-apoptotic. There is a significant decrease in cell viability observed in non-preconditioned *(NP)* uterine myocytes as compared to controls *(C)* and preconditioned *(P)* (n=3 per condition), when exposed to a known cytotoxic dose of Thaps (250nM, 1hr), 48 hrs post Thaps preconditioning (10nM, 24hrs). **(B)** At 48hrs to recovery there is equal activation of GRP78 and (**C)** Cl CASP3 in both P and NP uterine myocytes. (**D)** In contrast, Cl PARP is significantly decreased in the P versus NP cells. PDIA2 and NCOA3 are utilized as our cytoplasmic and nuclear protein loading controls. A representative blot from this experiment is shown. Statistical comparisons were performed using one-way ANOVA, and subsequent Newman-Keuls multiple-comparison tests. Data labeled with different letters are significantly different from each other (p<0.05).

**SFigure 4**


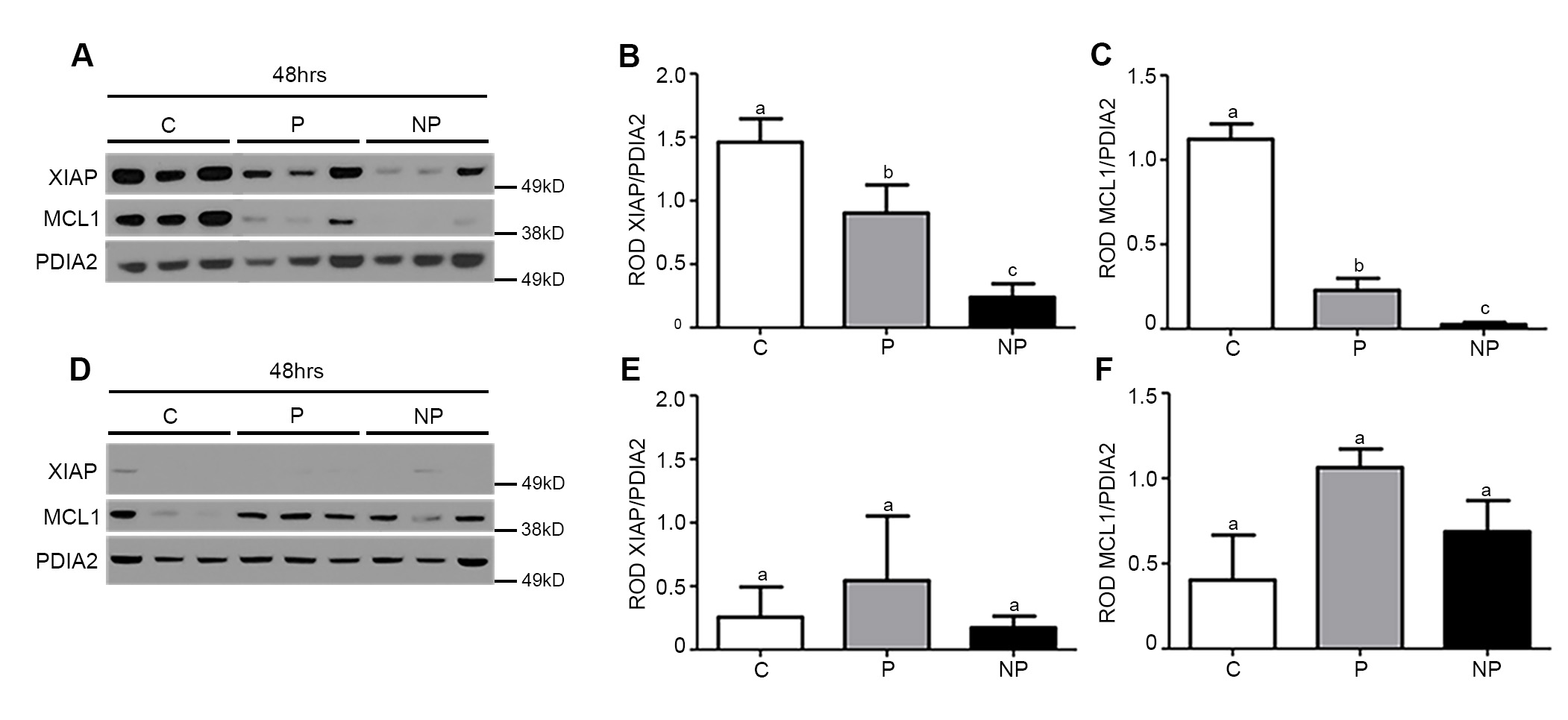


**Fig. S4.** Increased maintenance of pro-survival molecules with TM mediated UPR preconditioning in the human uterine myocyte**. (A)** Pro-survival molecules XIAP and Mcl-1 were analyzed 48hrs post TM-bolus in non-preconditioned *(NP),* TM-preconditioned cells given a 48hr recovery period *(P)* and vehicle treated controls *(C)*. (**B) and (C)** Both Mcl-1 and XIAP were significantly elevated in TM-preconditioned cells when compared to non-preconditioned myocytes. (**D)** The experiment was repeated using Thaps as a preconditioning and bolus stimuli. **(E)** and **(F)** Neither Mcl-1 or XIAP were significantly different between Thaps-preconditioned and non-preconditioned myocytes. A representative blot from this experiment is shown. PDIA2 is utilized as our cytoplasmic loading control. Statistical comparisons were performed using one-way ANOVA, and subsequent Newman-Keuls multiple-comparison tests. Data labeled with different letters are significantly different from each other (p<0.05).

**SFigure 5**


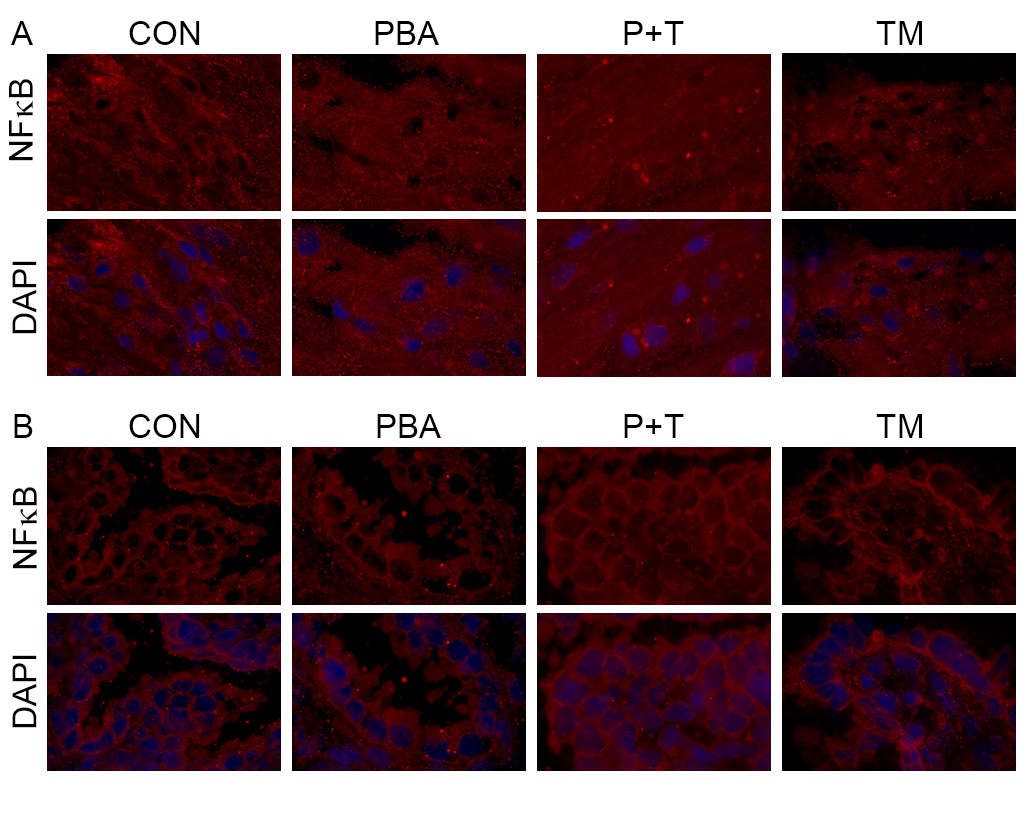


**Fig. S5.** Endogenous preconditioning prevents premature activation of NFκB in the myometrial and endometrial compartments of the pregnant mouse. Uteri collected from vehicle treated (*Con*), sub-preconditioned (*PBA*), exogenously stressed sub-preconditioned (*TM+PBA*) and exogenously stressed preconditioned (*TM*) mice on E17 prior to the onset of preterm or term birth were examined for activation of NF-κB in the **(A)** myometrium and **(B)** endometrial compartments via immunohistochemistry. Heightened NFκB activation was observed in both the **(A)** myometrium and **(B)** endometrium of the *TM+PBA* uteri.

**SFigure 6**

**
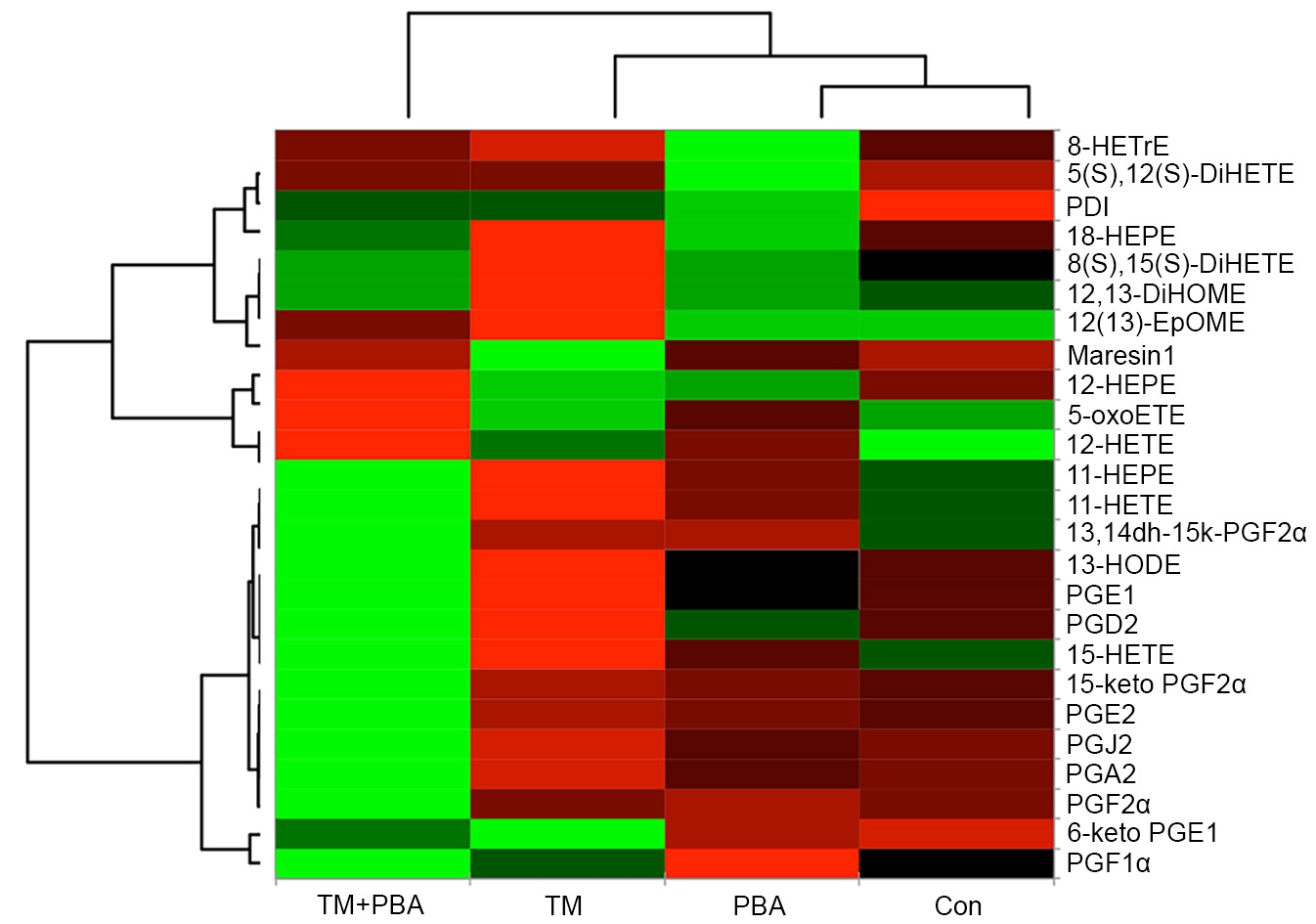
**

**Fig. S6.** UPR Preconditioning in vivo suppresses local uterine prostaglandin production. Uteri collected from vehicle treated (Con), sub-preconditioned (PBA), exogenously stressed sub-preconditioned (TM+PBA) and vehicle treated (TM) mice on E17 prior to the onset of preterm and term birth, were examined for prostaglandin levels. Significantly elevated levels of PGE1, PGE2, PGD3 were isolated to the sub-preconditioned mice exposed to a minor exogenous stress (TM+PBA). Furthermore downstream products of arachidonic acid metabolism were also significantly elevated in stressed-sub-preconditioned mice compared to preconditioned control.
